# Mutant C/EBPα p30 alleviates immunosuppression of CD8^+^ T cells by inhibiting autophagy‐associated secretion of IL‐1β in AML

**DOI:** 10.1111/cpr.13331

**Published:** 2022-09-20

**Authors:** Jun‐Dan Wang, Jue‐Qiong Xu, Xue‐Ning Zhang, Ze‐Wei Huang, Ling‐Ling Liu, Ling Zhang, Xin‐Xing Lei, Man‐Jie Xue, Jian‐Yu Weng, Zi‐Jie Long

**Affiliations:** ^1^ Department of Hematology, The Third Affiliated Hospital Sun Yat‐sen University Guangzhou China; ^2^ Institute of Hematology Sun Yat‐sen University Guangzhou China; ^3^ Department of Hematology Guangdong Provincial People's Hospital, Guangdong Academy of Medical Sciences Guangzhou China; ^4^ Sun Yat‐sen University Cancer Center Guangzhou China; ^5^ Medical Research Center, The Third Affiliated Hospital, Sun Yat‐sen University Guangzhou China

## Abstract

**Objectives:**

Mutant C/EBPα p30 (mp30), the product of C/EBPα double mutations (DM), lacks transactivation domain 1 and has C‐terminal loss‐of‐function mutation. Acute myeloid leukaemia (AML) patients harbouring C/EBPα DM could be classified as a distinct subgroup with favourable prognosis. However, the underlying mechanism remains elusive.

**Materials and Methods:**

Autophagy regulated by mp30 was detected by western blot and immunofluorescence. Immune infiltration analysis and GSEA were performed to investigate autophagic and inflammatory status of AML patients from the GSE14468 cohort. Flow cytometry was applied to analyse T cell activation.

**Results:**

Mp30 inhibited autophagy by suppressing nucleus translocation of NF‐κB. Autophagy‐associated secretion of IL‐1β was decreased in mp30‐overexpressed AML cells. Bioinformatic analysis revealed that inflammatory status was attenuated, while CD8^+^ T cell infiltration was upregulated in C/EBPα DM AML patients. Consistently, the proportion of CD8^+^CD69^+^ T cells in peripheral blood mononuclear cells (PBMCs) was upregulated after co‐culture with mp30 AML cell conditional culture medium. Knock‐out of IL‐1β in AML cells also enhanced CD8^+^ T cell activation. Accordingly, IL‐1β expression was significantly reduced in the bone marrow (BM) cells of C/EBPα DM AML patients compared to the wildtype, while the CD8^+^CD69^+^ T cell proportion was specifically elevated.

**Conclusions:**

C/EBPα DM alleviates immunosuppression of CD8^+^ T cells by inhibiting the autophagy‐associated secretion of IL‐1β, which elucidated that repression of autophagy‐related inflammatory response in AML patients might achieve a favourable clinical benefit.

## Graphical abstract

Mp30 suppresses autophagy‐associated IL‐β secretion, which ultimately alleviates the immunosuppression of CD8^+^ T cells in the microenvironment, contributing to favourable prognosis of AML patients.

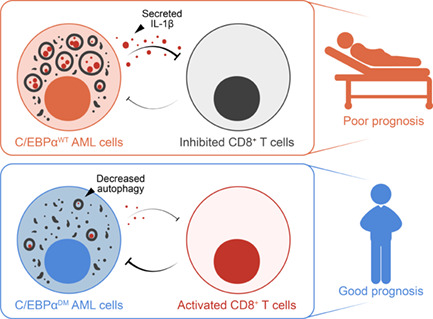

## 1 INTRODUCTION

Acute myeloid leukaemia (AML) is a clonal haematopoietic disease with myeloid differentiation blockage and haematopoietic function impairment. The incidence of AML is associated with chromosomal translocation or gene mutations, which have strong correlations with the response to clinic treatment and overall risk stratification.^1^

CCAAT‐enhancer‐binding protein α (C/EBPα) is a lineage‐specific transcription factor that involves in terminal differentiation in multiple types of cells. Alternative translational initiation attributes to two start sites within the same open reading frame. The first translational start site results in a full‐length 42‐kD C/EBPα protein (p42), while the second ATG translates a 30‐kD C/EBPα protein (p30).^2^ C/EBPα mutations are detected in approximately 10% AML patients.^3^ These mutations are clustered in either N or C terminal part of C/EBPα gene. N‐terminal mutations mostly harbour a frame shift in the open reading frame, expressing dominant p30, while C‐terminal mutations are in the basic leucine zipper domain.^4^ Most C/EBPα mutant AML patients harbour a combination of both N‐ and C‐terminal mutations locating in different alleles, also known as C/EBPα double mutations (DM), which ultimately lead to the absence of wildtype (WT) full‐length version, with the translation of p30, mutant p42 and mutant p30.^5^, ^6^ AML patients with C/EBPα DM are defined as a subgroup with favourable outcome when exposed to conventional chemotherapy.^7^ Nevertheless, the underlying mechanism has not been fully elucidated.

Autophagy and inflammation interplay in pro‐ and anti‐tumorigenesis. Numerous studies have shown that C/EBPα participates in autophagy process. In hepatic stellate cells, acetylated‐C/EBPα elevates its binding capacity to autophagy‐related protein Beclin1.^8^ Additionally, in hepatocellular carcinoma, C/EBPα could protect against energy starvation by improving autophagy‐involved lipid metabolism.^9^ Decline of C/EBPα triggers the expression of aberrant NADH dehydrogenase genes and enhances autophagy in chronic obstructive pulmonary disease.^10^ Based on these results, mutant C/EBPα may also involve in the occurrence of autophagy.

Importantly, autophagy is associated with inflammation. IL‐1β and IL‐18 production, and intestinal inflammation are observed in mice lacking Atg16L1 in haematopoietic cells.^11^ Indeed, inflammatory cytokine signalling leads to haematological disorder. IL‐6, IL‐1β, TNF‐α and GM‐CSF could accelerate the growth of AML cells.^12^, ^13^ In a murine model of colon cancer, lack of IL‐6 slows tumour growth and increase the infiltration of CD8^+^ T cells.^14^ Similarly, IL‐1β deficiency in murine breast cancer model significantly represses the tumour progression while enhancing activated CD8^+^ lymphocytes.^15^ Thus, inflammatory cytokines may inhibit T cell infiltration, providing potential targets for cancer therapy.

Here, we showed that mp30 inhibited NF‐κB nucleus translocation and suppressed cell autophagy represented by the reduction of LC3B II in AML cells. Moreover, mp30 decreased autophagy‐associated IL‐1β secretion and further reversed the suppression of CD8^+^ T cells in the microenvironment. Consistently, we further confirmed that AML patients harbouring C/EBPα DM were in a hypoinflammatory state with the recovery of T cell activation. Our finding demonstrated that suppression of inflammation might provide a therapeutic strategy to AML patients.

## 2 MATERIALS AND METHODS

### 2.1 Cell culture

HL‐60 and U937 cells were cultured in Roswell Park Memorial Institute (RPMI) 1640 medium (Gibco) supplemented with 10% foetal bovine serum (FBS, Gibco). 293FT cells were cultured in Dulbecco's modified Eagle's medium (DMEM, Gibco) supplemented with 10% FBS.

Bone marrow cells (BMs) or peripheral blood mononuclear cells (PBMCs) were obtained from healthy donors or untreated AML patients (Table S1) at the Department of Hematology. The study was approved by the Institute Research Ethics Committee at The Third Affiliated Hospital of Sun Yat‐sen University.

### 2.2 Cell proliferation assay

AML cells were cultured in a volume of 100 μl per well in a 96‐well plate with RPMI 1640 medium containing 10% FBS. Then 10 μl Cell Counting Kit‐8 reagent (APExBIO) was added to the medium and incubated for another 4 h followed by optical density measurement.

### 2.3 Plasmid construction and lentivirus infection

Mutant C/EBPα p30 (mp30) with a deletion of lysine at 313aa (937_939del) was inserted into pSin‐EF2‐Puro lentiviral vector. NF‐κB and IL‐1β knock‐out plasmids were generated by ligating sgRNAs to lenti‐CRISPR VII vector. 293FT was co‐transfected with corresponding vectors and packaging vectors using lipofectamine 2000 transfection reagent (Invitrogen) for lentivirus production. Supernatants were collected 48 h after transfection and subsequently used for infection. Cells were further sorted by puromycin. The sgRNA sequences used were listed in Table S2.

### 2.4 Conditional culture medium collection

An equal number of cells were plated in 24 well plates. After centrifugation, the supernatant of culture system was collected for conditional culture.

### 2.5 Western blot assay

Cells were lysed in RIPA buffer (Cell Signaling Technology, CST) containing protease inhibitor on ice for 15 min. Proteins were dispersed by sodium dodecyl sulphate‐polyacrylamide gel electrophoresis (SDS‐PAGE) and transferred to nitrocellulose membranes (Merk Millipore). The membranes were then blocked with 5% BSA at room temperature (RT) for 1 h, subsequently incubated with primary antibodies [C/EBPα (Zen‐Bioscience), NF‐κB (CST), phosphorylated‐IκB (CST), IκB (CST), Histone H3 (CST), LC3B (NOVUS), GAPDH (Santa Cruz), α‐tubulin (CST), IL‐1β (Proteintech)] and corresponding secondary antibodies conjugated with horseradish peroxidase (HRP). Immunoreactive bands were monitored by chemiluminescent imaging system (Tanon Science & Technology) with chemiluminescence reagent (Merk Millipore).

### 2.6 qRT‐PCR assay

Total RNA was extracted by TRIzol reagent (Invitrogen). The cDNA was obtained via a Reverse Transcription System (Invitrogen), and qRT‐PCR was conducted using Roche LightCycler 480 (Roche) with a PerfectStart Green qPCR SuperMix (TransGen Biotech). The primer sequences were shown in Table S3.

### 2.7 PBMCs isolation and culture

PBMCs were obtained using Ficoll solution following the manufacturer's instruction. For T cell activation assay, PBMCs were cultured in the conditional culture medium for 24 h followed by stimulated with 2.5 μg/ml CD3 and 0.5 μg/ml CD28 antibodies for 12 h.

### 2.8 Flow cytometry

Cells were collected, washed with PBS twice and resuspended into single cell suspension, and then incubated with CD69 (BioLegend), CD8 (BioLegend), TIM‐3 (CD366, BioLegend), LAG‐3 (CD223, BioLegend), or PD‐1 (CD279, BD Pharmingen) antibody for 30 min on ice. For measuring apoptosis, cells were incubated with Annexin V‐FITC and PI in the binding buffer for 15 min as recommended by the manufacturer (Vazyme). Multicolour flow cytometry analysis was performed by BD FACSCanto instrument. Data was analysed with Flowjo_V10 software.

### 2.9 Immunofluorescence staining

Cells were washed twice with PBS, and fixed in 4% paraformaldehyde. The fixed cells were permeabilized with 0.1% Triton X‐100, blocked with 1% BSA for 30 min, then incubated with NF‐κB (CST), LC3B (NOVUS), or IL‐1β (Proteintech) antibody O/N at 4°C. After three times washing in PBS, fluorescein‐labelled secondary antibodies were applied for 1 h at RT, and then cells were stained with DAPI for nuclei visualization.

### 2.10 LysoTracker Red‐staining

The content of lysosomes was evaluated by LysoTracker Red‐staining under manufacturer's instruction. In brief, cells were incubated with 75 nmol/L LysoTracker Red (Beyotime) for 1 h at 37°C. After washing with PBS, cells were observed under an inverted fluorescence microscope and analysed by the ImageJ software.

### 2.11 Acridine orange staining

293FT cell was transfected with mp30 or empty vector. After starving in EBSS for 3 h, cells were washed with PBS and stained with 1 μg/ml acridine orange for 15 min. Subsequently, cells were visualized under a fluorescence microscope.

### 2.12 Bioinformatics analysis

GSE14468 dataset was obtained from the Gene Expression Omnibus (GEO, https://www.ncbi.nlm.nih.gov/geo/), which included gene expression profiling of 514 AML cases including 26 C/EBPα DM versus 488 C/EBPα WT. Gene Ontology (GO), Kyoto Encyclopedia of Genes and Genomes (KEGG) and Gene set enrichment analysis (GSEA) were performed by the clusterProfiler package.^16^ For immune infiltration analysis, xCell algorithm was used to score the relative abundance of immune cells by the ‘xCell’ package.^17^ R version 4.1.0 software was used to conduct all of the analysis and data plotting. Gene expression matrix of C/EBPα DM and WT AML samples was applied to Monocle 2 (http://cole-trapnell-lab.github.iomonocle‐release/) for the trajectory analysis. Autophagy score was assessed based on Gene set variation analysis (GSVA).

### 2.13 Transwell migration assay

Cell migration assay was performed by 24‐well Boyden chamber (Corning). Briefly, conditional culture medium was placed in the lower chamber, while PBMCs were cultured with RPMI1640 with 10% FBS in the transwell inserts. PBMCs in the lower chamber were then collected and analysed by flow cytometry after 24 h.

### 2.14 Statistical analysis

Statistical significance was assessed using the Student's *t*‐test or Wilcoxon test by GraphPad Prism 8.0 software.

## 3 RESULTS

### 3.1 Mp30 reduces autophagy in AML cells

**FIGURE 1:**
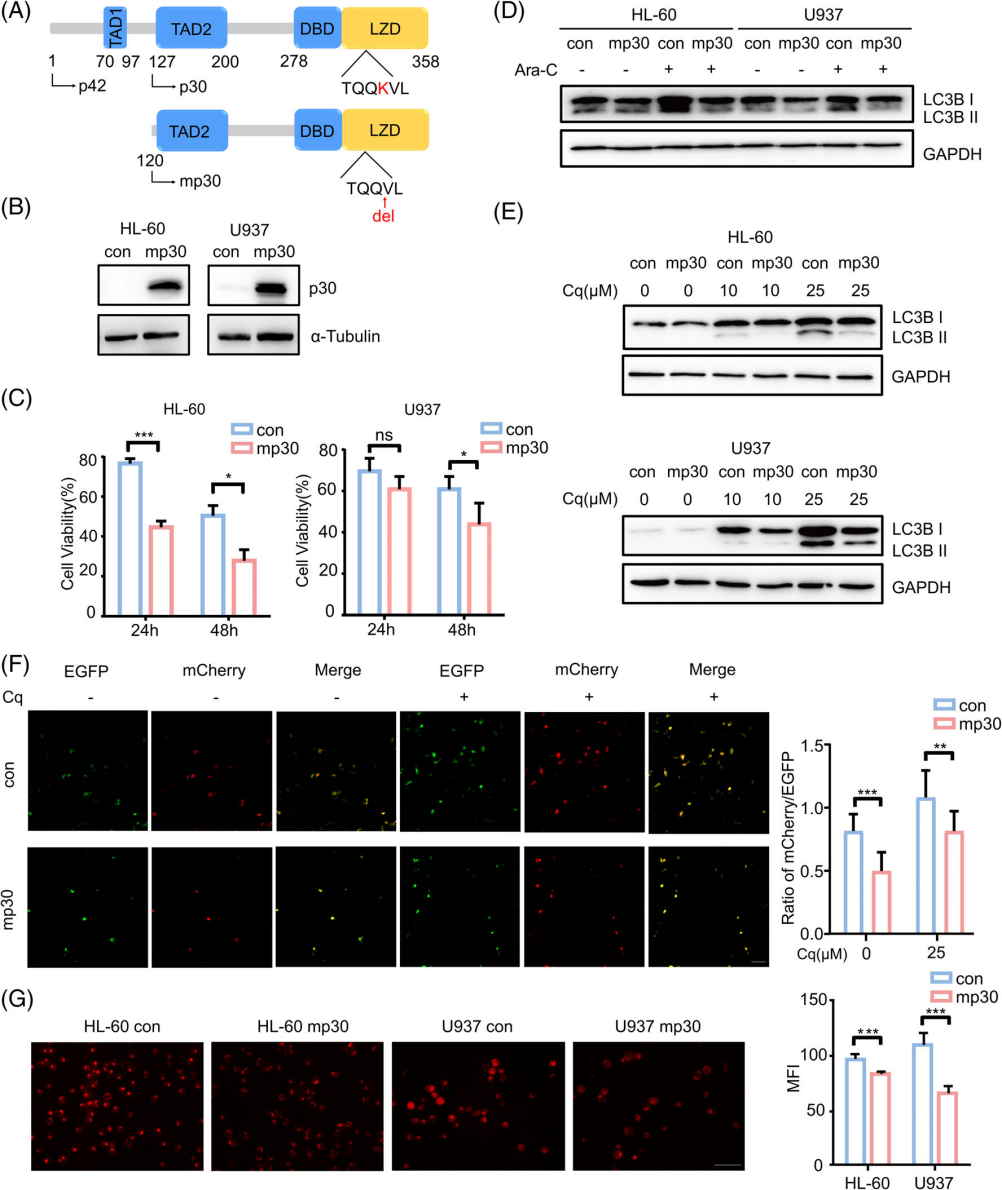
Mp30 reduces autophagy in AML cells. (A) Schematic presentation of C/EBPα wildtype and mp30 protein. (B) HL‐60 and U937 were infected with lentivirus which carried mp30 or counterpart control and subjected to western blot analysis of mp30 expression. (C) Cell viability was detected by CCK‐8 assay after exposing to 0.5 μM (HL‐60) or 0.2 μM (U937) Ara‐C. (D) LC3B expression was detected by western blot assay after exposing to 0.5 μM (HL‐60) or 0.2 μM (U937) Ara‐C. (E) HL‐60 and U937 cells were treated with 10 μM or 25 μM Cq and subjected to western blot analysis of LC3B lipidation. (F) 293FT cells were transfected with a mCherry‐EGFP tandem‐tagged LC3B vector and autophagic flux was determined as the ratio of mCherry to EGFP‐positive puncta (bar = 100 μm). (G) Lysosomal analysis of AML cells was measured by LysoTracker Red staining (bar = 50 μm). Data were shown as mean ± SD. *p < 0.05, **p < 0.01, ***p < 0.001, ns *p* ≥ 0.05

To find out the function of mp30 in AML, we constructed mp30‐overexpressed HL‐60 and U937 cells by lentivirus transfection (Figure 1A,B). Mp30 significantly sensitized HL‐60 and U937 to Ara‐C treatment (Figure 1C). Meanwhile, mp30 suppressed the long‐term but not short‐term proliferation (Figure S1A, S1B). Since tumour cells tended to acquire resistance to chemotherapeutic drugs by modulating autophagy, we next explored the correlation between the expression of mp30 and autophagy in AML cells. By exposing AML cells to Cytarabine (Ara‐C), we noted that mp30‐overexpressed cells induced less LC3B II expression compared to the control (Figure 1D). We further measured autophagy capacity in mp30‐overexpressed AML and 293FT cells. Both immunoblotting and fluorescence analysis showed that the lipidation of LC3B was significantly decreased in mp30‐overexpressed cells when exposed to chloroquine (Cq) (Figure 1E,F). Additionally, LysoTracker Red‐staining analysis also revealed that the content of lysosomes in mp30‐overexpressed cells was reduced (Figure 1G). Hence, mp30 could restrain autophagy in AML cells. Accordingly, inhibition of autophagy by Cq synergized with Ara‐C to suppress HL‐60 and U937 cell proliferation (Table S4).

### 3.2 Autophagy restrained by mp30 is mediated by suppression of NF‐κB nucleus translocation

**FIGURE 2:**
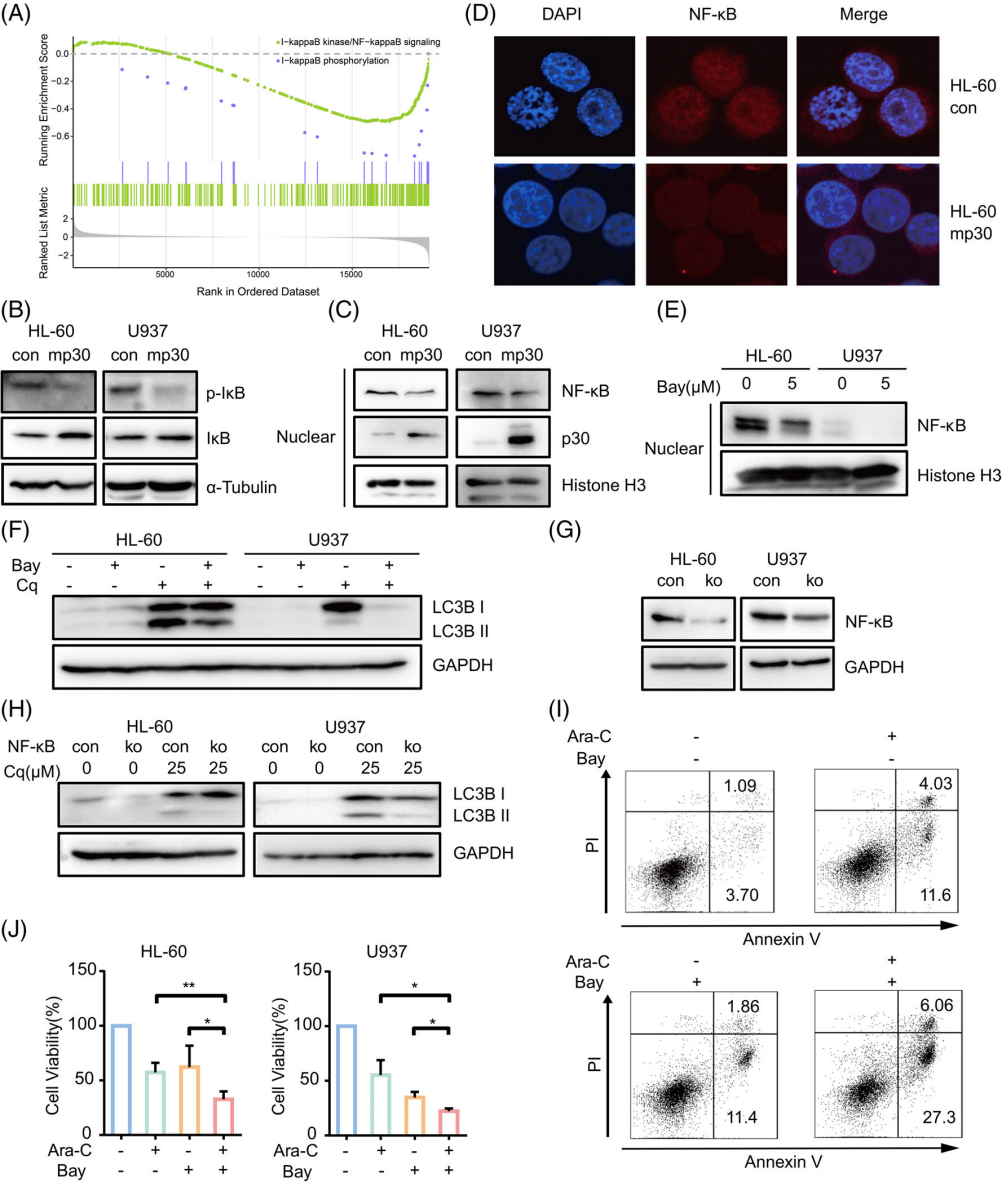
NF‐κB mediates autophagy downstream of mp30 in AML cells. (A) GSEA‐GO analysis between C/EBPα WT and DM samples (GSE14468). (B) Western blot analysis of p‐IκB and IκB expression in HL‐60 con/mp30 cells and U937 con/mp30 cells. (C) Western blot analysis of NF‐κB and mp30 expression in nuclear protein of control or mp30‐overexpressed AML cells. (D) HL‐60 control and mp30‐overexpressed cells were stained with NF‐κB antibody and DAPI. Representative data were shown. (E) HL‐60 and U937 cells were treated with or without 5 μM Bay 11‐7082 and subjected to western blot analysis for NF‐κB expression. (F) Cells were treated with or without 5 μM Bay 11‐7082 and/or 25 μM Cq, and subjected to western blot analysis of LC3B expression. Cells with NF‐κB knock‐out were treated with or without 25 μM Cq and subjected to western blot analysis of NF‐κB (G) and LC3B (H) expression. (I) HL‐60 cells were exposed to 0.5 μM Ara‐C alone or combined with 2 μM Bay 11‐7082. Apoptosis was measured by flow cytometry. (J) Cell viability was measured by CCK‐8 assay after exposing to 0.5 μM (HL‐60) or 0.2 μM (U937) Ara‐C alone or combined with 2 μM Bay 11‐7082. Data were shown as mean ± SD. *p < 0.05, **p < 0.01

We then investigated the underlying mechanism of mp30‐regulated autophagy in AML cells. We grouped GSE14468 cohort based on C/EBPα mutation and subsequently performed GSEA analysis. Intriguingly, we found that NF‐κB signalling, which had been shown to promote autophagy, was downregulated in C/EBPα DM group (Figure 2A). The activation of NF‐κB starts with a release from phosphorylated IκB in the cytosol, followed by translocating into nucleus.^18^ Mp30 reduced IκB phosphorylation in HL‐60 and U937 (Figure 2B). Meanwhile, NF‐κB nucleus translocation was decreased (Figure 2C,D). Moreover, immunoblotting assay indicated that Bay 11‐7082, an inhibitor of NF‐κB, could significantly reduce the translocation of NF‐κB into nucleus in HL‐60 and U937 cells (Figure 2E), accompanied by reduction of LC3B lipidation (Figure 2F). Consistently, CRISPR/cas9‐based knock‐out of NF‐κB also reduced LC3B lipidation (Figure 2G,H). These results indicated that NF‐κB suppression was involved in mp30‐downregulated autophagy. Since mp30 could sensitize HL‐60 and U937 to Ara‐C treatment, we therefore exposed AML cells to Ara‐C combined with Bay 11‐7082 to assess whether combined utilization could similarly sensitize AML cells without C/EBPα mutation. Result showed Bay 11‐7082 could enhance the apoptotic effect of Ara‐C and lead to proliferation blockage (Figure 2I,J). Thus, NF‐κB promoted autophagy in AML cells and suppression of NF‐κB might achieve therapeutic benefit.

### 3.3 C/EBPα DM diminishes inflammatory status and enhances CD8 ^+^ T cell activation in AML patients

**FIGURE 3:**
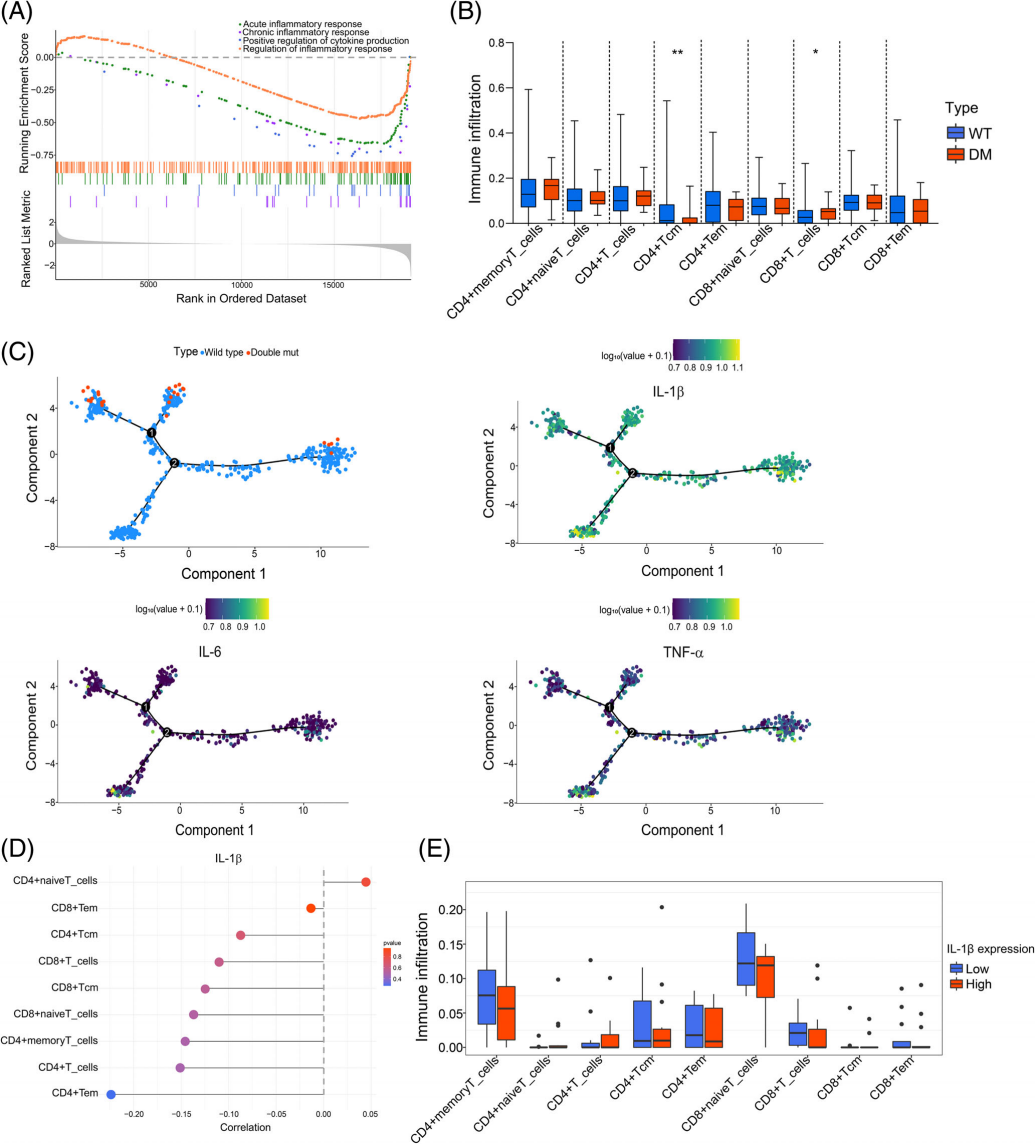
Bioinformatic analysis is performed in AML patients. (A) GSEA‐GO analysis between C/EBPα WT and DM samples (GSE14468). (B) T cell infiltration analysis between C/EBPα WT and DM samples. (C) Trajectory analysis of C/EBPα mutation status (top‐left), as well as IL‐1β (top‐right), IL‐6 (bottom‐left) and TNF‐α (bottom‐right) expression in AML patients using Monocle 2. Numbers on the trajectory line indicated branching points. (D) Correlation analysis between expression of IL‐1β and T cell infiltration was displayed in C/EBPα DM samples. (E) C/EBPα DM patients were categorized into two groups based on the expression of IL‐1β. Immune infiltration analysis between high and low IL‐1β groups was performed. *p < 0.05, **p < 0.01

We next characterized the difference of immune landscape between C/EBPα WT and DM AML patients in GSE14468. The result displayed that AML patients carrying C/EBPα DM were in an attenuated inflammatory status (Figure 3A). To identify the difference of T cell status between C/EBPα WT and DM samples, immune infiltration of T cells was analysed. It turned out that the CD8^+^ T cells were significantly increased in C/EBPα DM AML patients (Figure 3B). Thus, C/EBPα DM patients were in a hypoinflammation status with enhanced T cell activation. Previous studies have shown that pro‐inflammatory cytokines including IL‐1β, IL‐6 and TNF‐α are involved in the regulation of leukaemic blast formation.^12^ We therefore explored the relevance between each pro‐inflammatory cytokine and C/EBPα mutations. A trajectory analysis was applied to verify transitions in transcriptomic programs during AML progression in patients. To our surprise, IL‐1β, IL‐6 and TNF‐α expression were remarkably followed with the status of patients with C/EBPα DM or WT. C/EBPα DM patients displayed low expression of IL‐1β, IL‐6 and TNF‐α (Figure 3C). Thus, C/EBPα WT or DM samples were grouped according to expression of IL‐1β, IL‐6 and TNF‐α, respectively. Strikingly, IL‐1β was the most significant inflammatory factor that negatively correlated with T cell infiltration both in C/EBPα DM or WT patients (Figure 3D, S2A). Next, C/EBPα DM or WT patients were divided into IL‐1β expression high and low groups respectively. Immune infiltration analysis also revealed that there were more infiltrated CD8^+^ T cells in AML patients with low expression of IL‐1β both in C/EBPα DM or WT patients (Figure 3E, S2B). Taken together, IL‐1β might be one of the most important regulatory factors to trigger immune response in C/EBPα DM AML.

### 3.4 Downregulation of IL‐1β by mp30 alleviates immunosuppression of CD8 ^+^ T cells

**FIGURE 4:**
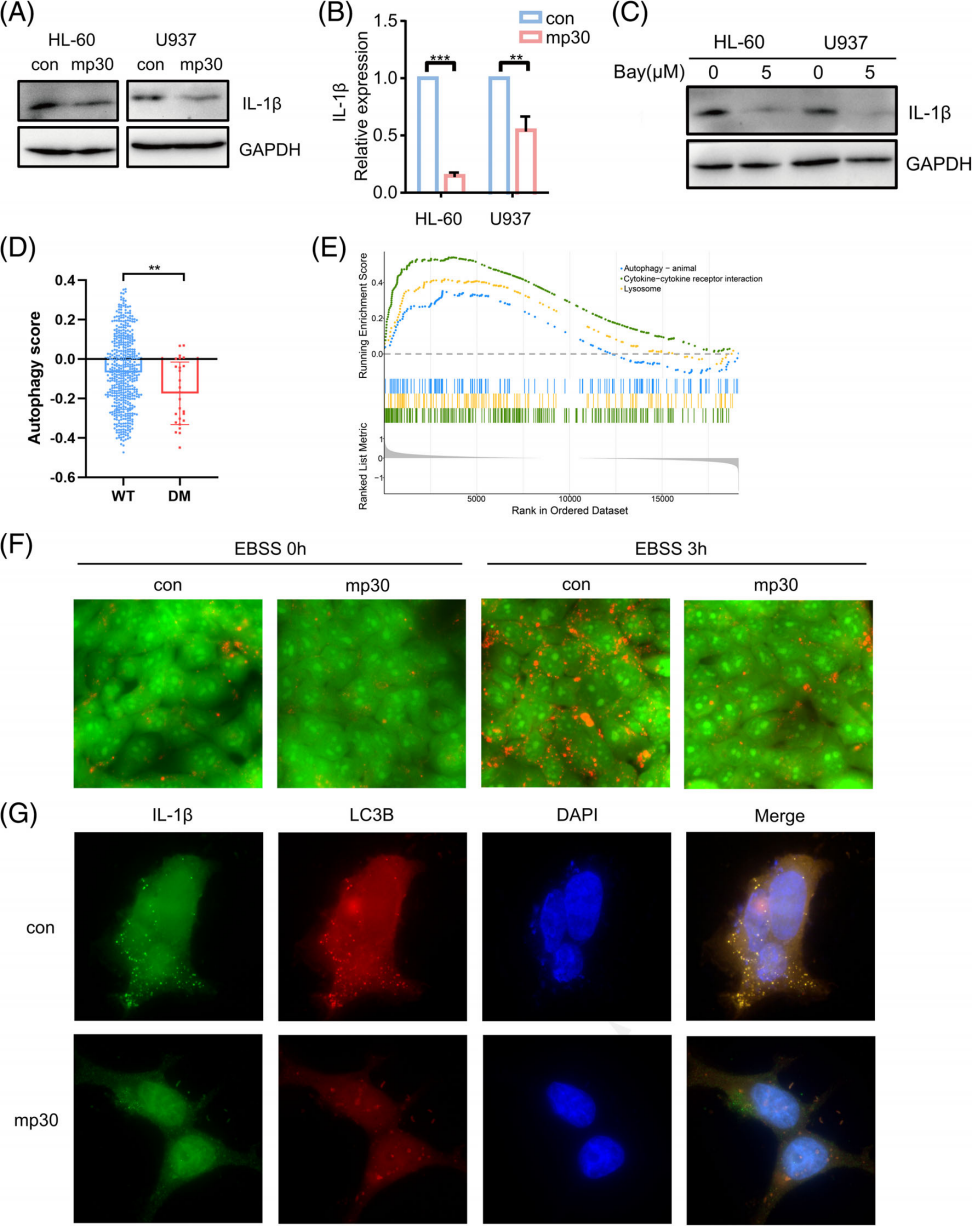
Mp30 decreases IL‐1β secretion in AML cells. IL‐1β expression was measured in HL‐60 and U937 cells with mp30 overexpression by western blot (A) and qRT‐PCR (B). (C) IL‐1β expression was measured by western blot in HL‐60 and U937 after 5 μM Bay 11‐7082 treatment for 24 h. (D) Autophagy score was accessed by GSE14468. (E) GSEA‐KEGG analysis between IL‐1β high and low groups in C/EBPα DM patients (GSE14468). (F) 293FT cells transfected with mp30 and control vector were starved in EBSS for 3 h and stained with 1 μg/ml acridine orange, and images were recorded under fluorescence microscopy (400×). (G) 293FT cells transfected with mp30 or control vector were stained with LC3B and IL‐1β antibodies after starved in EBSS, and images were recorded under fluorescence microscopy (1000×). Data were shown as mean ± SD. **p < 0.01, ***p < 0.001

To explore the regulation of mp30 on IL‐1β, we first evaluated IL‐1β expression by immunoblotting and qPCR, and noted that IL‐1β expression was significantly reduced by mp30 (Figure 4A,B). Similarly, Bay 11‐7082 treatment reduced IL‐1β expression (Figure 4C), further indicating this process was mediated by NF‐κB. By GSE14468 cohort, we found that autophagy score was lower in the C/EBPα DM group (Figure 4D). GSEA‐KEGG also revealed that in C/EBPα DM patients, autophagy, lysosome and cytokine‐cytokine receptor interaction signalling were enriched in high IL‐1β group (Figure 4E). This was supported by the study that IL‐1β secretion is mediated by autophagy.^19^ Additionally, acidic vacuoles were significantly detected in 293FT cells starved in EBSS for 3 h, indicating that autophagy could be induced by EBSS. However, mp30‐overexpressed cells displayed less autophagy phenotype (Figure 4F). Next, fluorescence microscopy analysis displayed the co‐localization of IL‐1β and LC3B was significantly decreased in mp30‐overexpressed 293FT cells after starvation (Figure 4G). These results suggested mp30 not only decreased IL‐1β expression but also reduced its secretion by reducing IL‐1β incorporated into the autophagosome.

**FIGURE 5:**
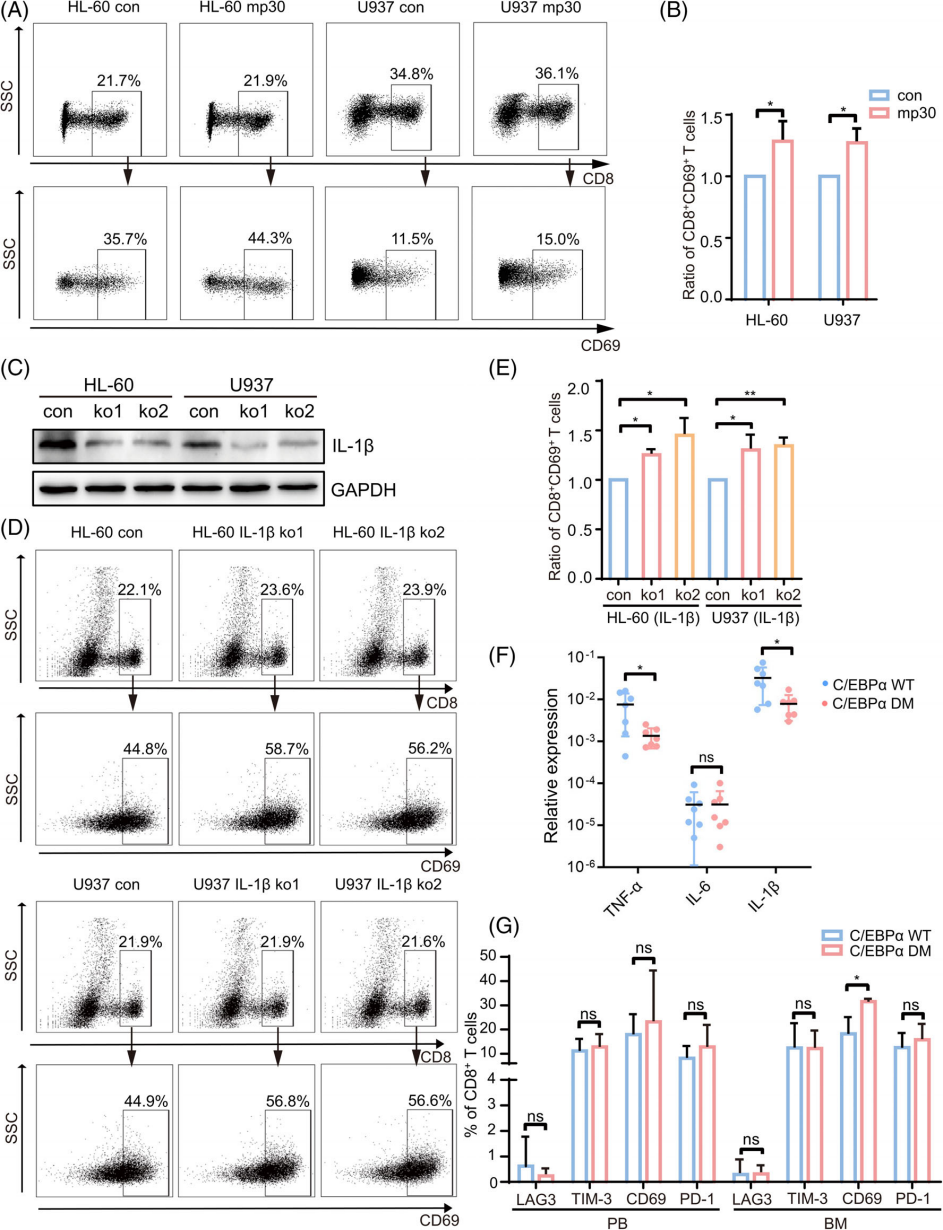
C/EBPα DM tends to alleviate immunosuppression of CD8^+^ T cells with reduced IL‐1β expression. PBMCs were cultured in conditional culture medium from AML cells for 24 h followed by anti‐CD3/CD28 stimulation for 12 h and then subjected to flow cytometry analysis. CD69 was considered as an indicator of CD8^+^ T cell activation. The representative data was displayed in (A) and the statistical analysis (HL‐60, *n* = 5; U937, *n* = 3) was shown in (B). (C) Western blot was used to detect the knock‐out efficacy of IL‐1β in HL‐60 and U937 cells. PBMCs were cultured in conditional culture medium from IL‐1β knock‐out AML cells for 24 h followed by anti‐CD3/CD28 stimulation for 12 h and then subjected to flow cytometry analysis. The representative data was displayed in (D) and the statistical analysis (*n* = 3) was shown in (E). (F) qRT‐PCR analysis of the relative mRNA expression of IL‐1β in control (*n* = 7) and C/EBPα DM (*n* = 7) AML patients. (G) CD8^+^ T cell activation and exhaustion markers were analysed by flow cytometry from both peripheral blood (PB) and bone marrow (BM) in C/EBPα WT (*n* = 4) and C/EBPα DM (*n* = 4) AML patients. Data were shown as mean ± SD. *p < 0.05, **p < 0.01, ns *p* ≥ 0.05

We subsequently explored whether IL‐1β secretion could change the interaction between AML cells and immune cells. Conditional culture medium from mp30‐overexpressed AML cells elevated migration capacity of CD8^+^ T cells in PBMCs derived from healthy donors (Figure S3). Meanwhile, by culturing PBMCs in culture medium, we noted that the proportion of CD69^+^ cells was also elevated in CD8^+^ T cells (Figure 5A,B). We then knocked out IL‐1β in HL‐60 and U937 cells and found that downregulation of IL‐1β significantly reversed the suppression of CD8^+^ T cells after co‐culture (Figure 5C–E). We further confirmed that IL‐1β mRNA expression was significantly lower in the bone marrow (BM) cells of C/EBPα DM AML patients than WT (Figure 5F), while the CD8^+^CD69^+^ T cell proportion was specifically elevated in BM rather than peripheral blood (PB). Concurrently, other T cell exhaustion markers have no statistical difference between two groups (Figure 5G). These results suggested that mp30 improved the function of CD8^+^ T cells by reducing IL‐1β production in C/EBPα DM AML.

## 4 DISCUSSION

AML originates from aberrant haematopoietic progenitors with the accumulation of multiple acquired genetic lesions, which results in myeloid differentiation blockage and eventually haematopoietic impairment. Gene mutation and chromosomal translocation have been indicated to occur in the early stage of leukaemia and may provide selective advantages for the clonal expansion of leukaemic stem cells and final progression of AML.^1^, ^20^

C/EBPα functions in the stage from common myeloid progenitors (CMPs) to granulocyte/monocyte progenitors (GMPs) for haematopoietic differentiation.^21^ Multiple studies have explored the correlation between C/EBPα mutations and prognosis in AML, which consistently supports a conclusion that patients harbouring C/EBPα DM tend to have favourable outcomes.^7^, ^22^, ^23^ The product of certain C/EBPα N‐terminal mutations drive a dominant‐negative effect on wildtype C/EBPα protein and inhibit granulocytic differentiation.^24^ Both aberrant granulopoiesis and haematological malignancy are also observed in a mouse model lacking C/EBPα p42, indicating expression of C/EBPα p30 alone could lead to unchecked proliferative capacity and increasing self‐renewal ability of myeloid progenitors.^25^ Thus, C/EBPα p30 may play as an oncogene in AML progression. Since studies just focus on C/EBPα C‐terminal mutations with functional loss of DNA binding capacity, we wondered whether mutant C/EBPα p30 might dominate the better outcome of C/EBPα DM. Our data showed that mutant C/EBPα p30 sensitized AML cells to Ara‐C treatment and regulated immune status via suppressing autophagy‐associated IL‐1β secretion.

Previous studies have shown that dysfunction of autophagy is involved in leukaemia progression and drug resistance. Certain oncogene mutation, such as KIT^D816V^, induces autophagy to promote AML development and maintenance.^26^ Similarly, in murine myeloid leukaemia, ablation of ATG5, the key autophagy protein, could increase apoptosis of differentiated malignant myeloid cells and prolong the survival of MLL‐AF9‐driven AML mice.^27^ Autophagy often promotes leukaemia cell proliferation or survival by preventing apoptosis or delaying necrosis. Prosurvival autophagy leads to drug resistance in AML LSCs exposed to JQ1, a bromodomain and extraterminal domain (BET) inhibitor.^28^ In t (8;21) AML cells, the addition of autophagy inhibitor, such as chloroquine, synergizes with HDAC inhibitor to induce cell death via accumulating ubiquitinated protein.^29^ Additionally, Atg7 inhibition in AML cells could overcome stroma‐mediated chemo‐resistance.^30^ Of note, inhibition of autophagy in some cancer types have already been employed in clinical treatment.^31^ Previous studies have already demonstrated the correlation between autophagy and C/EBPα.^8^, ^9^, ^10^ However, whether mutant C/EBPα could regulate autophagy in AML has not been clearly reported. After being treated with or without chloroquine, mp30‐overexpressed cells showed reduced accumulation of LC3B II and lysosomal content compared to control group (Figure 1E–G), revealing that mp30 could suppress autophagy in AML cells. In addition, mp30 significantly sensitized AML cells to Ara‐C treatment (Figure 1C). These findings implied that autophagy inhibition might contribute to Ara‐C sensitivity in mp30‐overexpressed AML cells.

NF‐κB‐activated inflammatory system is associated with tumour development and progression.^32^ The molecules link between NF‐κB‐activated inflammation and tumour promotion has been proposed, such as IL‐1β, IL‐6 and TNF‐α, which are downstream targets of NF‐κB. In AML, disease progression promoted by NLRP3 inflammasome is mediated by IL‐1β and NF‐κB signalling.^33^ An gel electrophoresis mobility shift assay with an oligonucleotide probe corresponding to an NF‐κB DNA‐binding motif of the IL‐1β gene promoter reveals that NF‐κB activation is the positive regulator of inflammatory cytokine gene expression during monocytic cell differentiation.^34^ In this study, we confirmed NF‐κB signalling was downregulated in C/EBPα DM samples compared to C/EBPα WT by GSEA analysis (Figure 2A). NF‐κB could enhance cell autophagy by inducing autophagy‐related proteins such as Beclin1, p62 or others.^18^ Similarly, we found that inhibition of NF‐κB by Bay 11‐7082 or knocking out NF‐κB significantly suppressed autophagy and reduced LC3B lipidation (Figure 2F,H). However, whether NF‐κB directly binds to IL‐1β to induce its transcription or indirectly improves IL‐1β expression and secretion by autophagy need to be further investigated.

Bioinformatic analysis indicated that patients with C/EBPα DM were in a low‐grade state of inflammation with enhanced T cell infiltration (Figure 3A,B). Since there is a strong correlation between chronic inflammation and tumour progression,^35^ dysregulation of cytokines production might lead to the poor prognosis of AML patients without C/EBPα DM. IL‐1β is a proinflammatory factor that has been described as hyperexpressed in various types of solid cancers as well as leukaemia.^36^ IL‐1β signalling promotes the expansion of leukaemia cells while suppressing normal myeloid progenitors.^13^ A previous study has also demonstrated that an autophagy‐associated secretion of IL‐1β in non‐macrophage cells.^37^ Of note, autophagy plays an essential role in unconventional secretion of cytosolic proteins. Autophagy secreted proteins, such as IL‐1β, CXCL8, LIF, are shown to be elevated in high‐autophagy melanoma cell lines.^38^ Cancer‐associated fibroblasts promote metastasis of non‐small cell lung cancer cells by autophagic secretion of HMGB1 via NF‐κB signalling.^39^ HMGB1 also promotes the synthesis of pro‐IL‐1β in THP‐1 macrophages by NF‐κB activation.^40^ Indeed, the secretion of IL‐1β is accompanied with autophagy since IL‐1β and LC3B co‐localize in the cytoplasm.^19^ In our study, we noted that the subcellular colocalization of IL‐1β and LC3B were reduced in mp30‐overexpressed 293FT cells after starvation, suggesting that mp30 decreased autophagy‐associated IL‐1β secretion (Figure 4G).

Importantly, high T cell percentage in the bone marrow is positively correlated with leukaemia‐free survival in newly diagnosed AML patients.^41^ And CD8^+^ T cells derived from AML patients are accompanied with an increased expression of exhaustion molecules.^42^ Moreover, loss of tumour cell‐derived IL‐1β signalling in tumour stroma significantly enhances CD8^+^ cytotoxic T cell infiltration and activation followed by attenuating pancreatic tumour growth, suggesting CD8^+^ T cells are the key component with antitumor immunity.^43^ Thereby, we investigated the impact on CD8^+^ T cells after mp30 overexpression. Recruitment and activation of CD8^+^ T cells were shown to be promoted by mp30 (Figure 5A,B and S3), which was consistent with the favourable outcome of patients harbouring C/EBPα DM. We further verified that the mRNA expression of IL‐1β was significantly downregulated in patients with C/EBPα DM compared to WT (Figure 5F), which was consistent with the finding of mp30 in the cell model. Meanwhile, CD8^+^ T cell activation was elevated in the bone marrow of C/EBPα DM AML patients rather than in peripheral blood (Figure 5G). These results indicated that inflammation was restrained in AML patients harbouring C/EBPα DM, and suppression of IL‐1β could relieve the immune suppression. Thus, IL‐1β might play as a negative downstream factor of mp30‐mediated favourable outcome.

Therefore, our finding demonstrated that mp30 inhibited autophagy‐associated IL‐1β secretion followed by CD8^+^ T cell activation. For clinical treatment, restraining inflammation might provide a novel strategy for AML therapy.

## AUTHOR CONTRIBUTIONS

Jun‐Dan Wang, Jue‐Qiong Xu, Xue‐Ning Zhang, Ze‐Wei Huang, Xin‐Xing Lei, and Man‐Jie Xue performed experiments and analysed the data. Ling‐Ling Liu and Ling Zhang collected the clinical samples. Zi‐Jie Long and Jian‐Yu Weng performed study design and revised the manuscript. All authors read and approved the final manuscript.

## CONFLICT OF INTEREST

The authors declare that they have no competing interests.

## Supporting information


**Figure S1** Proliferation effect of mp30‐overexpressed AML cells. (A) Short‐term proliferation was detected in mp30 cells and control cells at 24 h and 48 h by CCK‐8 assay. (B) Colony formation assay was performed to measure the long‐term proliferation ability of mp30 cells. After 7 days, colony number was counted while diameter was measured by Image J. Data were shown as mean ± SD. ***p < 0.001, ns *p* ≥ 0.05.

**Figure S2** Bioinformatic analysis of wildtype AML patients. (A) Correlation analysis between expression of inflammatory factor and T cell infiltration was displayed in C/EBPα WT samples. (B) C/EBPα WT samples were categorized into quartiles based on the expression of IL‐1β. Immune infiltration analysis between high (upper quartile) and low (lower quartile) IL‐1β expression groups was performed. *p < 0.05, ***p < 0.001, ns *p* ≥ 0.05.

**Figure S3** Recruitment of CD8^+^ T cells co‐cultured with C/EBPα mp30 AML cell conditional culture medium. Migration assays were indicated by transwell with PBMCs in upper chamber and conditional culture medium from different groups in lower chamber. After 24 h, cells in lower chamber were collected and analysed (HL‐60, *n* = 4; U937, *n* = 3). Data were shown as mean ± SD. *p < 0.05, **p < 0.01.

**Table S1** Clinical and molecular characteristics of AML patients.

**Table S2** sgRNA used for NF‐κB and IL‐1β knock‐out.

**Table S3** Primers used for qRT‐PCR.

**Table S4** Synergistic effects of Cytarabine (Ara‐C) and Chloroquine (Cq) in HL‐60 and U937 cells.

## Data Availability

Main data generated or analysed during this study are included in this article, and detailed data are available from the corresponding authors on reasonable request.
