# Supplementary figures and images for: Mutant C/EBPα p30 alleviates immunosuppression of CD8+ T cells by inhibiting autophagy‐associated secretion of IL‐1β in AML
Source: Cell Prolif. 2022 Sep 20;55(12):e13331. doi: 10.1111/cpr.13331 (PMC9715362; doi:10.1111/cpr.13331)

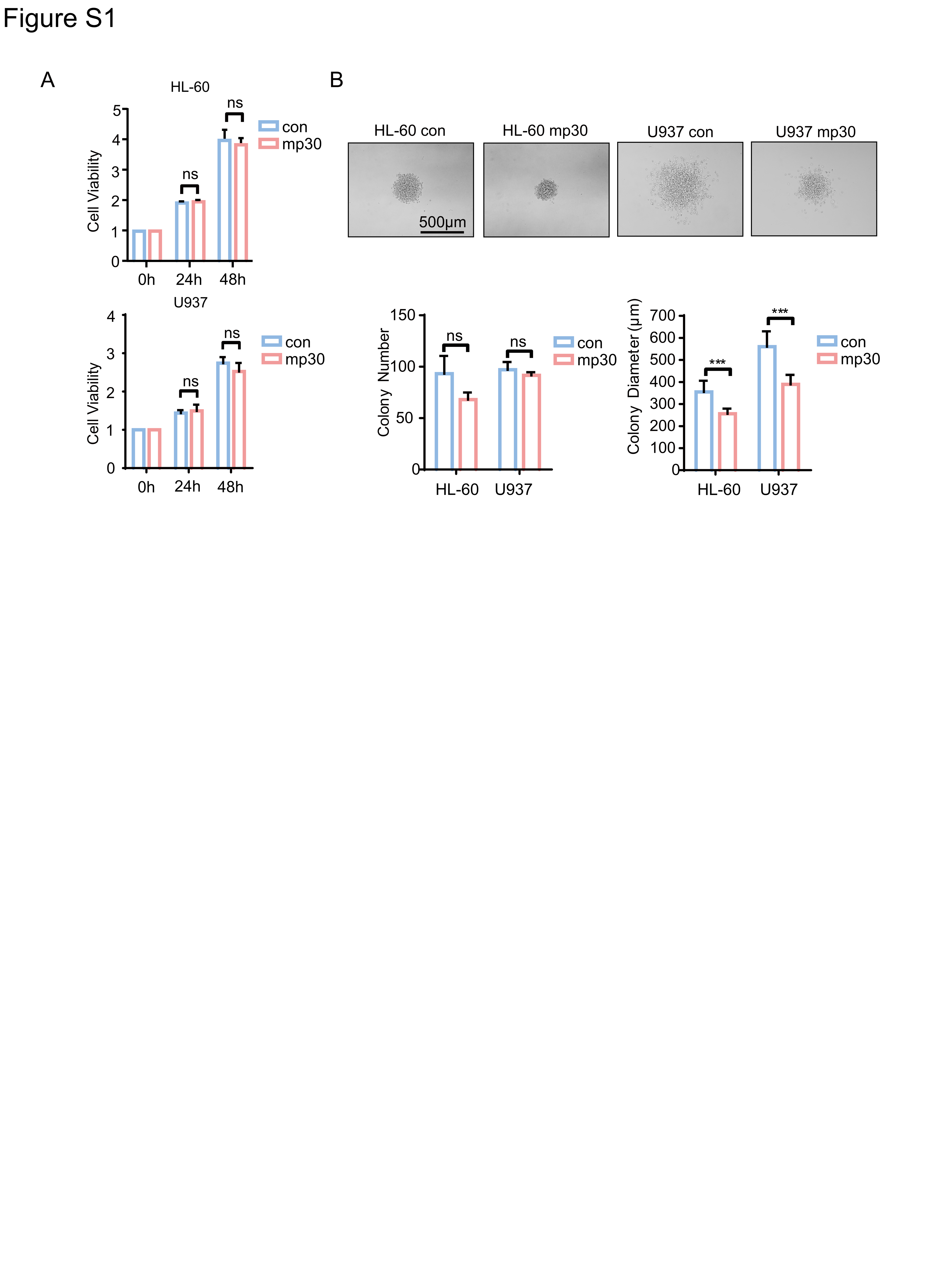

Supplement: Supplementary file 1 — Figure S1 Proliferation effect of mp30‐overexpressed AML cells. (A) Short‐term proliferation was detected in mp30 cells and control cells at 24 h and 48 h by CCK‐8 assay. (B) Colony formation assay was performed to measure the long‐term proliferation ability of mp30 cells. After 7 days, colony number was counted while diameter was measured by Image J. Data were shown as mean ± SD. ***p < 0.001, ns p ≥ 0.05. [file CPR-55-e13331-s002.tif]

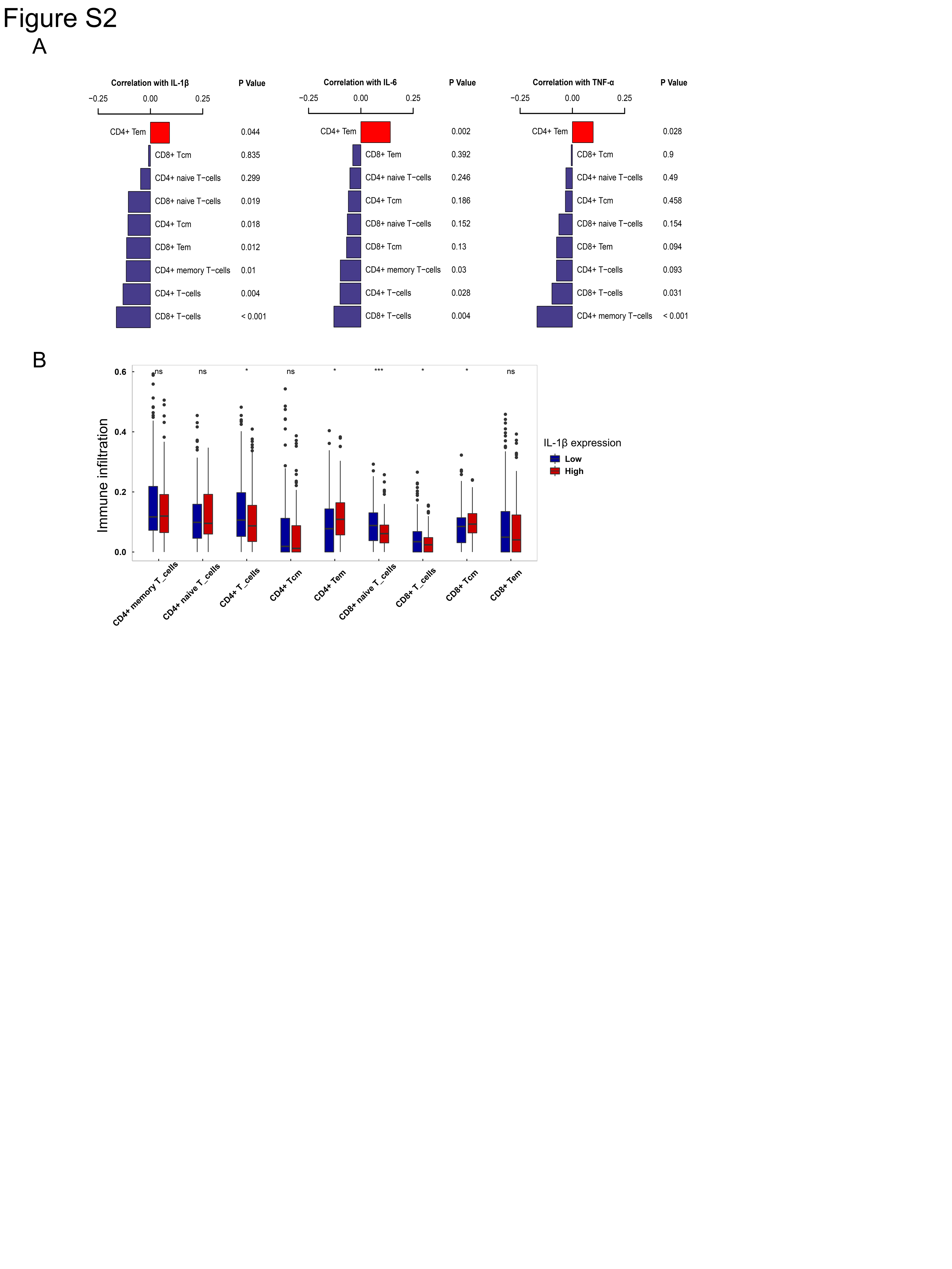

Supplement: Supplementary file 2 — Figure S2 Bioinformatic analysis of wildtype AML patients. (A) Correlation analysis between expression of inflammatory factor and T cell infiltration was displayed in C/EBPα WT samples. (B) C/EBPα WT samples were categorized into quartiles based on the expression of IL‐1β. Immune infiltration analysis between high (upper quartile) and low (lower quartile) IL‐1β expression groups was performed. *p < 0.05, ***p < 0.001, ns p ≥ 0.05. [file CPR-55-e13331-s004.tif]

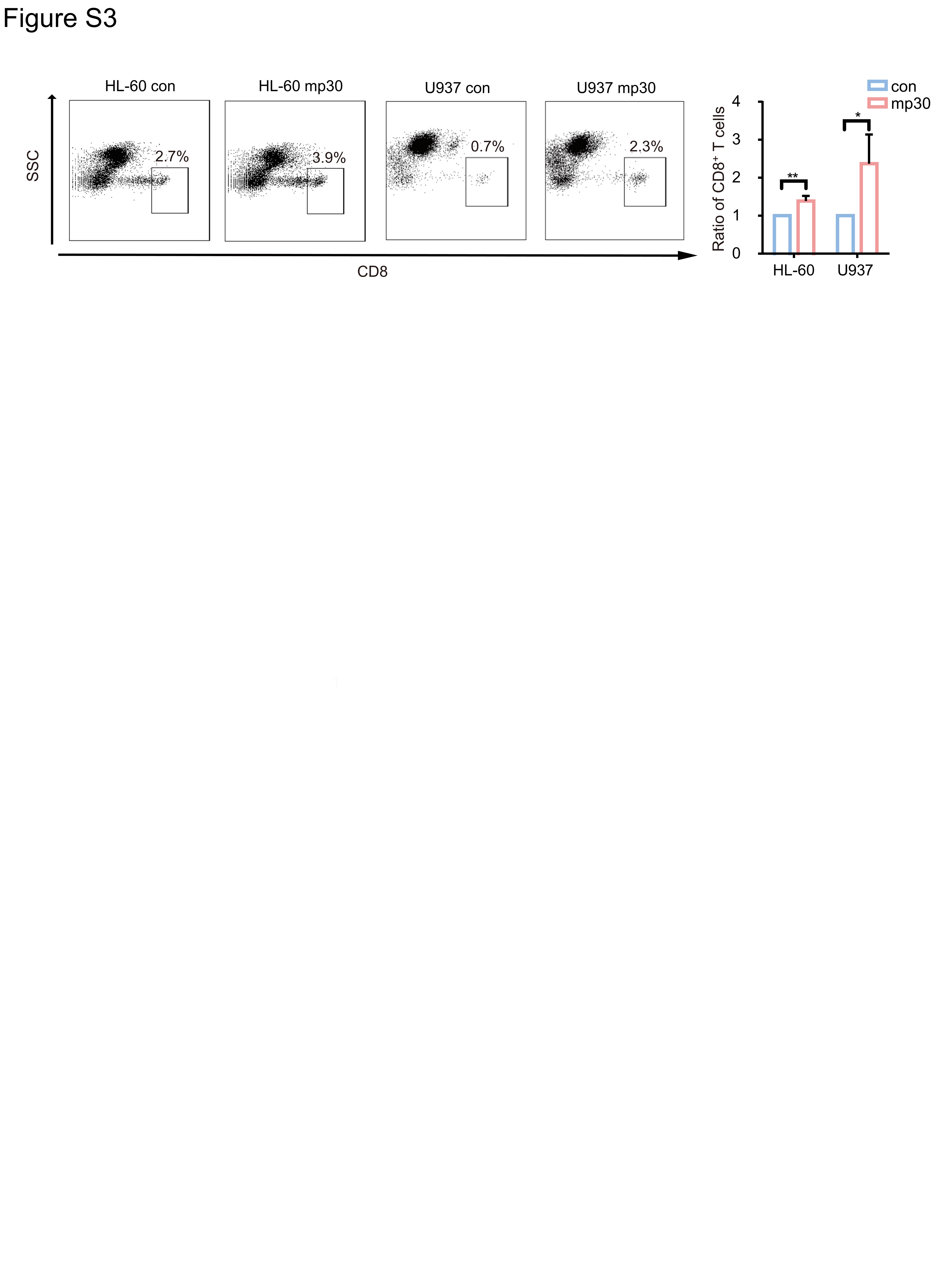

Supplement: Supplementary file 3 — Figure S3 Recruitment of CD8+ T cells co‐cultured with C/EBPα mp30 AML cell conditional culture medium. Migration assays were indicated by transwell with PBMCs in upper chamber and conditional culture medium from different groups in lower chamber. After 24 h, cells in lower chamber were collected and analysed (HL‐60, n = 4; U937, n = 3). Data were shown as mean ± SD. *p < 0.05, **p < 0.01. [file CPR-55-e13331-s001.tif]
