# Supplementary material for: Mutant C/EBPα p30 alleviates immunosuppression of CD8+ T cells by inhibiting autophagy‐associated secretion of IL‐1β in AML
Source: Cell Prolif. 2022 Sep 20;55(12):e13331. doi: 10.1111/cpr.13331 (PMC9715362; doi:10.1111/cpr.13331)
Supplement: Supplementary file 4 — Table S1 Clinical and molecular characteristics of AML patients. [file CPR-55-e13331-s003.docx]

**Table S1. Clinical and molecular characteristics of AML patients.**

| **No.** | **Age**  **/Gender** | **WBC**  **(×10^9^/L)** | **C/EBPα**  **Double Mutation** | **Other mutation** |
| --- | --- | --- | --- | --- |
| 1 | 85/M | 3.47 | Yes | KRAS, BRCA2, IKZF1, KMT2D, SH2B3 |
| 2 | 44/M | 61.1 | Yes | WT1, MPL, DDX41, TERT |
| 3 | 16/M | 2.79 | Yes | NRAS, JAK3 |
| 4 | 28/M | 0.38 | Yes | GATA2, RAD21, CBL |
| 5 | 57/F | 2.44 | Yes | NRAS, GATA2 |
| 6 | 37/F | 3.22 | Yes | NF1, EP300, GATA2, BRCA1, BRCA2, PHF6 |
| 7 | 27/F | 4.82 | Yes | EP300, NF1 |
| 8 | 35/F | 58.16 | No | FLT3, IDH2, NPM1, CBL, DNMT3A, KMT2D |
| 9 | 50/M | 4.64 | No | NF1,TP53 |
| 10 | 55/M | 82.97 | No | FLT, RUNX1, IDH1, EP300, GATA2, TAL1 |
| 11 | 57/M | 65.4 | No | BCOR, NPM1, CBL, FLT3, BRCA2, DNMT3A |
| 12 | 58/M | 9.04 | No | DNMT3A, NPM1, FLT3, KMT2A, PTPN11 |
| 13 | 51/M | 3.1 | No | CSF3R, KIT, BRCA1 |
| 14 | 65/F | 12.05 | No | BCOR, NRAS, RUNX1, SF3B1, RUNX1 |

Abbreviations: M, male; F, female; WBC, white blood cell.
