# Supplementary material for: Mutant C/EBPα p30 alleviates immunosuppression of CD8+ T cells by inhibiting autophagy‐associated secretion of IL‐1β in AML
Source: Cell Prolif. 2022 Sep 20;55(12):e13331. doi: 10.1111/cpr.13331 (PMC9715362; doi:10.1111/cpr.13331)
Supplement: Supplementary file 5 — Table S2 sgRNA used for NF‐κB and IL‐1β knock‐out. [file CPR-55-e13331-s005.docx]

**Table S2. sgRNA used for NF-κB and IL-1β knock-out.**

| **Target** | **sgRNA** |
| --- | --- |
| NF-κB Forward | 5’-CACCGCTCGTCTGTAGTGCACGCCG-3’ |
| NF-κB Reverse | 5’-AAACCGGCGTGCACTACAGACGAGC-3’ |
| IL-1β Forward 1 | 5’-CACCGCTTCGACACATGGGATAACG-3’ |
| IL-1β Reverse 1 | 5’-AAACCGTTATCCCATGTGTCGAAGC-3’ |
| IL-1β Forward 2 | 5’-CACCGGGTGGTCGGAGATTCGTAGC-3’ |
| IL-1β Reverse 2 | 5’-AAACGCTACGAATCTCCGACCACCC-3’ |
