# Supplementary material for: Mutant C/EBPα p30 alleviates immunosuppression of CD8+ T cells by inhibiting autophagy‐associated secretion of IL‐1β in AML
Source: Cell Prolif. 2022 Sep 20;55(12):e13331. doi: 10.1111/cpr.13331 (PMC9715362; doi:10.1111/cpr.13331)
Supplement: Supplementary file 6 — Table S3 Primers used for qRT‐PCR. [file CPR-55-e13331-s006.docx]

**Table S3. Primers used for qRT-PCR.**

| **Gene** | **Primer** |
| --- | --- |
| IL-1β Forward | 5’-ATGATGGCTTATTACAGTGGCAA-3’ |
| IL-1β Reverse | 5’-GTCGGAGATTCGTAGCTGGA-3’ |
| TNF-α Forward | 5’-GAGGCCAAGCCCTGGTATG-3’ |
| TNF-α Reverse | 5’-CGGGCCGATTGATCTCAGC-3’ |
| IL-6 Forward | 5’-ACTCACCTCTTCAGAACGAATTG-3’ |
| IL-6 Reverse | 5’-CCATCTTTGGAAGGTTCAGGTTG-3’ |
| ACTB Forward | 5’-CATGTACGTTGCTATCCAGGC-3’ |
| ACTB Reverse | 5’-CTCCTTAATGTCACGCACGAT-3’ |
