# Supplementary material for: Mutant C/EBPα p30 alleviates immunosuppression of CD8+ T cells by inhibiting autophagy‐associated secretion of IL‐1β in AML
Source: Cell Prolif. 2022 Sep 20;55(12):e13331. doi: 10.1111/cpr.13331 (PMC9715362; doi:10.1111/cpr.13331)
Supplement: Supplementary file 7 — Table S4 Synergistic effects of Cytarabine (Ara‐C) and Chloroquine (Cq) in HL‐60 and U937 cells. [file CPR-55-e13331-s007.doc]

**Table S4. Synergistic effects of Cytarabine (Ara-C) and Chloroquine (Cq) in HL-60 and U937 cells.**

|  | Ara-C(μM) | Cq(μM) | D_Ara-C_ | D_Cq_ | D_Ara-C+Cq_ | Q |
| --- | --- | --- | --- | --- | --- | --- |
| HL-60 | 0.5 | 10 | 0.27 | -0.19 | 0.39 | 2.95 |
|  | 0.5 | 20 | 0.27 | -0.03 | 0.43 | 1.76 |
| U937 | 0.5 | 10 | 0.34 | 0.00 | 0.50 | 1.44 |
|  | 0.5 | 20 | 0.34 | 0.10 | 0.48 | 1.16 |

Q= D_Ara-C + Cq_ / (D_Ara-C_ + D_Cq_ - D_Ara-C_ × D_Cq_)

D: Inhibition rate of treatment for 24h.
